# A patient with amyotrophic lateral sclerosis and atypical clinical and electrodiagnostic features: a case report

**DOI:** 10.1186/1752-1947-5-538

**Published:** 2011-11-02

**Authors:** Alexander Venizelos, Youngsook Park, Morris A Fisher

**Affiliations:** 1 Department of Neurology, Neurology (127), Hines VAH, Hines, IL 60141, USA; 2 Loyola University Chicago Medical Center, 2160 S. First Ave., Maywood, IL 60153, USA

## Abstract

**Introduction:**

Amyotrophic lateral sclerosis is a rapidly progressive, fatal neurodegenerative disorder for which there is no effective treatment. The diagnosis is dependent on the clinical presentation and consistent electrodiagnostic studies. Typically, there is a combination of upper and lower motor neuron signs as well as electrodiagnostic studies indicative of diffuse motor axonal injury. The presentation of amyotrophic lateral sclerosis, however, may be variable. At the same time, the diagnosis is essential for patient prognosis and management. It is therefore important to appreciate the range of possible presentations of amyotrophic lateral sclerosis.

**Case presentation:**

We present the case of a 57-year-old Caucasian man with pathological findings on postmortem examination consistent with amyotrophic lateral sclerosis but atypical clinical and electrodiagnostic features. He died after a rapid course of progressive weakness. The patient did not respond to immunosuppressive therapy.

**Conclusion:**

Amyotrophic lateral sclerosis should be considered in patients with a rapidly progressive, unexplained neuropathic process. This should be true even if there are atypical clinical and electrodiagnostic findings. Absence of response to therapy and the development of upper motor neuron signs should reinforce the possibility that amyotrophic lateral sclerosis may be present. Since amyotrophic lateral sclerosis is a fatal illness, however, the possibility of this disease in patients with atypical clinical features should not diminish the need for a thorough diagnostic evaluation and treatment trials.

## Introduction

Amyotrophic lateral sclerosis (ALS) is a progressive neurodegenerative disorder characterized by anterior horn cell and corticospinal degeneration, primarily involving motor neurons in the cerebral cortex, brainstem, and spinal cord. Despite its recognition for over 140 years, ALS remains a poorly understood, usually rapidly progressive and fatal disease. More than 60% of patients with ALS die within three years [1]. Riluzole is the only recognized medication treatment and its effect is modest, prolonging life for only two to three months [2]. Given this, an accurate diagnosis is critical. A primary responsibility for the physician caring for patients with ALS is considering other potentially treatable illnesses including an acquired neuropathy.

Although criteria are available for diagnosing ALS [3,4], the diagnosis may be difficult given the variability of clinical findings and absence of a biological marker. The importance of electrodiagnostic studies in making a diagnosis of ALS has recently been emphasized [3]. We describe he case of a patient who had clinical and electrodiagnostic features that would be unusual for ALS, and yet was found to have pathological findings indicative of ALS on post-mortem examination. This study emphasizes the limitations of using currently accepted clinical and electrodiagnostic criteria in diagnosing ALS.

## Case presentation

A 57-year-old Caucasian man with no known past medical history presented two months after the onset of bilateral lower extremity weakness. The weakness initially affected his right leg, with subsequent progression to his left leg. He then noted 'muscle twitching'. There had been no preceding illness or insect bites. For several months prior to his illness he had been painting his house and reportedly was exposed to mold. Examination showed mild decrease in strength at the hips and knees with a somewhat more pronounced decrease at the ankles and toes, more prominent on the right side than the left. Strength in the arms was preserved. Fasciculations were observed in the arms and legs. Initial sensory exam revealed decreased position in the toes and decreased vibration in the legs. Cerebellar examination was unremarkable. Reflexes were present and symmetrical except for absent Achilles' reflexes. He was unable to stand from sitting without using his arms for support, nor able to walk unassisted. Electrodiagnostic studies (initial; see below) revealed findings consistent with a polyneuropathy, possibly multifocal. Laboratory testing (see below) did not reveal a cause for the patient's difficulties. An elevated protein in the cerebrospinal fluid was thought consistent with an acquired neuropathy. He was given a presumptive diagnosis of a chronic acquired demyelinating polyneuropathy (CIDP) and received a course of intravenous immunoglobulin (0.4 g/kg over four days) without symptomatic relief. He was then started on prednisone (60 mg daily) and azathioprine (150 mg daily). Computed tomography (CT) scans of the chest and abdomen were unremarkable as was a gallium scan. MRI scans of the entire spine were unrevealing except for some mild degenerative changes in the lumbar region. His weakness worsened with progressive decreased movement in the legs as well as weakness in the arms associated with atrophy in intrinsic hand muscles. He was admitted for further workup. A left sural nerve biopsy revealed findings of a chronic axonal neuropathy with active Wallerian degeneration and remyelination without evidence of inflammation. The patient was treated with plasmapheresis (equivalent of one plasma volume five times in 14 days) and discharged to a nursing home on prednisone and azathioprine. He was non-ambulatory. Five months later he returned to the clinic with worsening of his lower extremity weakness. Otherwise, his examination was the same except that reflexes were now brisk without spread and persistently absent Achilles' reflexes. Babinski signs were not present. A second electrodiagnostic examination five months after the initial study (repeat; see below) showed findings consistent with a progressive polyneuropathy. He continued to symptomatically deteriorate, and was admitted for intravenous cyclophosphamide. He died one month later of cardiorespiratory failure at age 58, seven months after symptom onset. There is no known family history of similar problems in a large well, known family. Electrodiagnostic studies (Table 1) were performed according to standard protocols used in the Clinical Neurophysiology Laboratories at the Hines Veterans Administration Hospital. F-waves were analyzed following 20 supramaximal stimuli. On needle electromyography (EMG) in the initial study, fibrillations and positive sharp waves were limited to the leg muscles with associated voluntary motor unit activation present distally. Fasciculations were present in the proximal arms and legs. The right tibial motor conduction velocity was in a demyelinating range and that for the right peroneal nerve was borderline [5]. There was a meaningful difference in conduction velocities between the right and left legs [6]. Distal motor latencies were diffusely prolonged in the feet, sensory conductions were slowed, and F-wave latencies in the median nerve in the arms were slowed despite unremarkable median motor conduction studies. A repeat examination three months later revealed findings that would be consistent with progression of a peripheral neuropathic process. Fibrillations and positive sharp waves were now present in the hands with associated decreased motor unit activation. Voluntary motor unit activation in the legs was absent distally and decreased proximally. Evoked motor responses were absent stimulating in the legs including recording from the tibialis anterior. The right median conduction velocity was in a demyelinating range with prominent prolongation of the median distal motor latency and median F-wave latency. There was meaningful asymmetry between the tested right median motor conduction velocity (29 m/sec) and that for the ulnar nerve (47 m/sec) [6]. There was no evidence for conduction block or temporal dispersion. Measurements for the motor conduction studies included negative peak and total durations as well as negative peak areas (Table 1). Recording sites in parentheses, median and ulnar Sensory conduction studies were performed orthodromically. Predicted F-wave latencies were based on regression equations including age and limb length [7].

**Table 1:** Electrodiagnostic Studies

		Initial						Repeat		
	
		Right			Left			Right		
**Study**	**Nerve**	**DML (ms)**	**AMP (mV)**	**CV (m/sec)**	**DML (ms)**	**AMP (mV)**	**CV (m/sec)**	**DML (ms)**	**AMP (mV)**	**CV (m/sec)**

**Motor**	*Tibial (AH)*	**6.6**	**0.4**	**26**	**7.4**	**1**	**37**	**NR**	**NR**	**NR**
	
	*Peroneal (EDB)*	**7.9**	**0.1**	**29**	**7.9**	**12**	**37**	**NR**	**NR**	**NR**
	
	*Median (APB)*	4.4	7	51				**6.4**	**0.7**	**29.6**
	
	*Ulnar (ADM)*	3.4	8	56				**3.5**	**2.4**	**47**

**Sensory**	*Sural*		21.2	**31**						
	
	*Median(dig 2)*		13	53					**3.6**	**44**
	
	*Median(dig 3)*		**9.4**	49						
	
	*Ulnar*		8.7	**46**					**3.0**	**42**

		**Mean F-wave latency (ms)**						**Mean F-wave latency (ms)**		

**F-Wave**	*Tibial(soleus)*	**NR**								
	
	*Median (APB)*	**33 **(predicted 28.9)						**41.4 **(predicted 28.9)		

*Normal values: *DML - peroneal ≤ 6.1 ms; tibial ≤ 6.4 ms; median ≤ 4.5 ms; ulnar ≤ 3.4 ms. MNCV (amplitudes) - tibial ≥ 39 m/sec (≥ 4 mV); peroneal ≥ 39 m/sec (≥ 3 mV); median ≥ 49 m/sec (≥ 4 mV); ulnar ≥ 49 m/sec (≥ 4 mV). SNAP conduction velocities (amplitudes) - sural ≥ 39 m/sec (≥ 6 μ); median ≥ 49 m/sec (10 μV); ulnar ≥ 49 m/sec (≥ 3 μV). Abnormal values in bold.

Extensive laboratory evaluations in this patient did not reveal a cause for his neuropathies. A complete blood count and a basic metabolic profile were all within normal limits as were studies of thyroid function and B12. A serum protein electrophoresis, prostate specific antigen, and urine for heavy metals were all unrevealing. This was also true for antibodies for Lyme disease and human immunodeficiency virus as well cultures for *campylobacter jejuni*. A complete SensoriMotor Neuropathy Profile and a NeoComplete Paraneoplastic Profile [Athena^R^, Worcester, MA] were unremarkable. These studies included assays for anti-GM1, anti-GD1, and myelin associated glycoprotein (MAG) antibodies as well as studies for Hu, Ma, and Yo antigens. Cerebrospinal protein was elevated (73 mg/dl; normal ≤ 45). There were no cells or oligoclonal bands.

**Figure 1:**
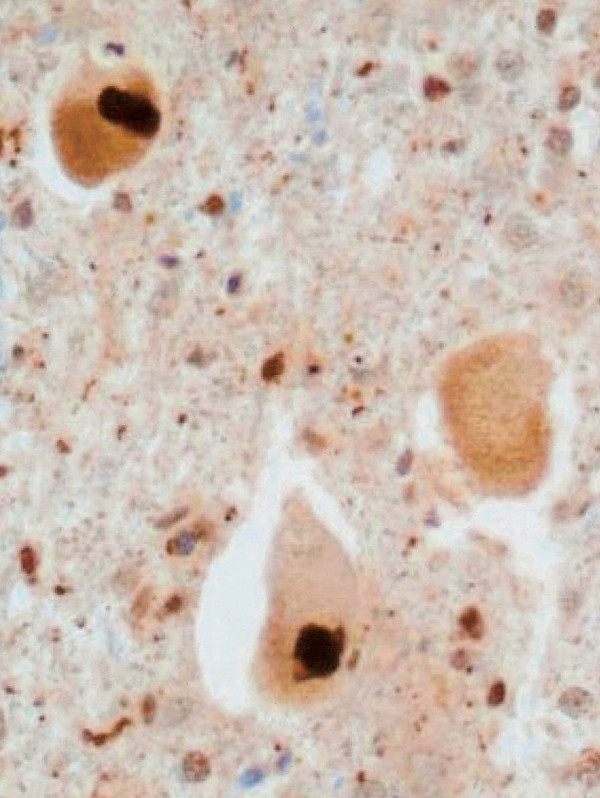
**Ubiquitin stain of the anterior horn neurons with Lewy body-like inclusions**. High powered field.

A post-mortem examination was obtained. There was loss of anterior horn cells, some with ubiquitin positive inclusions, and associated astrocytosis of the anterior horns throughout the entire length of the spinal cord. Lewy body-like (Figure 1) and skein-like inclusions were present. The lateral and anterior columns showed extensive vacuolar degeneration with macrophages and reactive gliosis. The posterior columns were preserved. The corticospinal tracts of the medulla were mildly vacuolated. The motor nuclei of the brainstem and cortex showed mild degenerative changes. There was no evidence of inflammation anywhere throughout the neuraxis.

Sections of peripheral nerve revealed loss of nerve fiber with extensive axonal degeneration and active Wallerian degeneration. There were a few CD26 positive B lymphocytes thought to be a nonspecific finding or a reaction of extensive degeneration. The muscle showed extensive atrophy with fiber type grouping and angulated fibers. There was no muscle inflammation. The final diagnoses from the autopsy were spinal cord showing extensive degeneration of anterior horn cells and tracts consistent with ALS and axonal degeneration of peripheral nerve.

## Discussion

When first seen, the patient had none of the requisite combination of upper and lower motor neuron signs consistent with ALS [3,4]. Although he subsequently developed brisk reflexes, at no time was there spread of the reflexes nor pathological reflexes such as Babinski responses. The asymmetry of leg weakness would be consistent with ALS. Asymmetric weakness, however, is also consistent with an acquired neuropathic process with demyelinating features [8,9]. Fasciculations were present and are a characteristic clinical finding in ALS. Fasciculations, however, are a non-specific finding related to neuropathic dysfunction. Fasciculations are not considered of clinical significance for ALS without associated evidence of neurogenic changes on needle EMG in the motor units recorded as fasciculations [10]. Such motor unit abnormalities were not seen. This patient had findings that could be consistent with a lower motor neuron variant of motor system disease, but the sensory loss would argue for a polyneuropathy. Sensory loss unless otherwise explained is considered inconsistent with a diagnosis of ALS [3].

More striking were the atypical electrodiagnostic findings for ALS. His electrodiagnostic studies contained all of those features that have been reported as rare or not present at all in ALS; namely, motor conduction velocities less than 70% of the lower limit of normal, distal motor latencies greater than 125% of the upper limit of normal, and F-wave latencies greater than 125% of the upper limit of normal [11]. Although a large number of criteria sets have been proposed for defining an acquired demyelinating neuropathy electrodiagnostically, none have really proven satisfactory [12]. Nevertheless, the pattern of abnormalities in this patient would be compatible with an acquired neuropathy with possible demyelinating features. Most striking were the asymmetries consistent with a multifocal process. Large differences in motor conduction velocities between his legs were observed on the initial electrodiagnostic examination, even allowing for differences in evoked response amplitudes. Also, in the repeat study, there were large differences between the median and ulnar nerves in the right arm, even allowing for the prolonged median distal motor latency [6]. Multifocal acquired demyelinating sensory and motor neuropathy (MADSAM; Lewis-Sumner syndrome) could be considered but characteristic conduction block was not present. In addition, the rapid progression with ultimate death in our patient would be inconsistent with previous reports of the natural history of MADSAM [13].

Three patients have recently been described who had a polyneuropathy resembling CIDP but were thought to have ALS [14]. Two patients had a family history of ALS. There was no family history of ALS in our patient. The only post mortem examination was performed on one of the patients with famalial ALS. The mean disease duration was 23 months with the shortest being 13 and the longest 38, considerably longer than in our patient. There have been two patients described in published manuscripts with multifocal motor neuropathy (MMN) and pathological changes of ALS [cited in reference [14]]. There was no evidence for MMN in our patient including absence of conduction block and GM1 antibodies and rapid course. Our patient is therefore arguably unique.

Electrodiagnostic studies in patients with possible ALS are critical for not only aiding in the diagnosis but also helping to provide information that could be consistent with a different, potentially treatable, diagnosis. A main consideration here would be a chronic acquired demyelinating polyneuropathy [15]. Treatment of these neuropathies can be costly and is not without risk. Studies such as electrodiagnosis that can provide justification for such treatments are therefore important. Our patient was treated as if he may have had a chronic acquired demyelinating neuropathy. Given the essentially invariably fatal outcome of ALS, treatment for CIDP was probably justified.

## Conclusions

This patient is a seemingly unique lesson on our limitations in making the distinction between ALS and an acquired, treatable neuropathy. Sensitivity to these issues is important given the serious nature of ALS and the resultant complexities in the management of such patients.

## Consent

Written informed consent was obtained from the patient's next-of-kin for publication of this case report and any accompanying images. A copy of the written consent is available for review by the Editor-in-Chief of this journal.

## Abbreviations

ADM: abductor digiti minimi; AH: abductor hallucis; AMP: amplitude; APB: abductor pollicis brevis; CV: conduction velocity; DML: distal motor latency; EBD: extensor digitorum brevis; NR: no response.

## Competing interests

The authors declare that they have no competing interests.

## Authors' contributions

All of the authors were involved in drafting and revising the manuscript and have given final approval of the report. AV and MAF made substantial contributions to the conception and design as well analysis and interpretation of data. MAF and YP made substantial contributions to the acquisition of the data.
